# Pregnancy outcomes and risk factors of serious cardiovascular adverse events in pregnant women with pulmonary hypertension

**DOI:** 10.1097/JS9.0000000000001016

**Published:** 2024-02-08

**Authors:** Yanming Kang, Xiaoqin Jiang

**Affiliations:** a Department of Anesthesiology, West China Second University Hospital, Sichuan University; b Key Laboratory of Birth Defects and Related Diseases of Women and Children (Sichuan University), Ministry of Education, Wuhou, Chengdu, China

*Dear Editor*:

A recent article published in the International Journal of Surgery mentioned that complication-specific risk assessment and personalized interpretation would provide a comprehensive and thorough understanding of surgical risk^1^, particularly for high-risk patients. Pulmonary hypertension (PH) in pregnant women is associated with increased rates of maternal and foetal mortality, resulting in recommendations to avoid conception^2,3^. The risk of PH deterioration is particularly high during the period around 32–34 weeks of pregnancy, immediately after delivery, and within the first 3 days postpartum, due to increased blood volume or a hypercoagulable state^4,5^. These factors can exacerbate pulmonary hypertension, precipitate right heart failure, and even trigger a pulmonary hypertension crisis. Although survival rates have significantly improved, cardiac complications remain frequent and severe, highlighting the importance of risk assessment and timely intervention to improve prognosis. This study aimed to investigate the clinical characteristics and pregnancy outcomes of women with PH, analyze the incidence of severe cardiovascular adverse events (SCAEs), and identify factors associated with SCAEs during pregnancy and the perinatal period (up to 6 weeks postpartum).

This study was approved by the Ethics Committee of West China Second Hospital (No. 2023/214). A retrospective analysis was conducted on data obtained from pregnant women with PH who underwent pregnancy termination at the hospital between August 2009 and January 2022. Information pertaining to antenatal examinations, cardiac evaluations, deliveries, and postpartum investigations was gathered from electronic medical records. SCAEs were defined as combined outcomes during pregnancy and the perinatal period, encompassing deteriorating cardiac function (NYHA class III or IV), severe arrhythmias, cardiac arrest, and mortality. Assessment of serious arrhythmias included ventricular arrhythmia, supraventricular arrhythmia, bradyarrhythmia, and QTc-interval prolongation. PH was diagnosed based on an estimated pulmonary artery pressure (PAP) exceeding 35 mmHg as determined by echocardiography.

In total, 127 pregnant women with increased PAP were included in the study. Of these, 109 pregnant women were included in the analysis (Fig. 1A, Supplementary Table 1, Supplemental Digital Content 1, http://links.lww.com/JS9/B824). Eighty-two (75.2%) pregnant women had less than 12 years of schooling (Supplementary Figure 1 A, Supplemental Digital Content 1, http://links.lww.com/JS9/B824). Forty-four (40.4%) pregnant women were diagnosed with pre-conceptional PH, 66 (60.6%) had congenital heart disease (Supplementary Figure 1 B, Supplemental Digital Content 1, http://links.lww.com/JS9/B824), and 55 (50.5%) were multipara, indicating their strong intention to continue having children. Additionally, 83 (76.2%) pregnant women were admitted to the hospital after 28 weeks of pregnancy, resulting in missed opportunities for early detection, appropriate intervention, and termination. The incidence of SCAEs was 50.5% (Supplementary Table 2, Supplemental Digital Content 1, http://links.lww.com/JS9/B824). The majority of patients (83.5%) were admitted to the ICU for an average monitoring and treatment period of 3 days. The average length of hospital stays for all patients was 8.0 days. The foetal and neonatal mortality rates were 10.1% and 0.9%, respectively. The rate of preterm birth, low birth weight, and neonatal ICU admission were 61.5%, 39.4%, and 40.4%, respectively.

**Figure 1:**
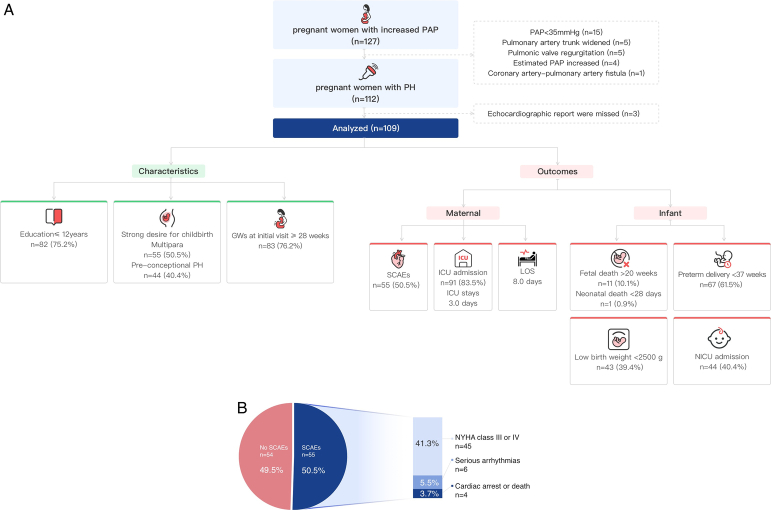
(A) Flow chart of the study. (B) The incidence of SCAEs in pregnant women with PH during pregnancy and the perinatal period. GWs, gestational weeks; LOS, the length of hospital stays; NICU, neonatal intensive unit; NYHA, New York Heart Association; PAP, pulmonary artery pressure; PH, pulmonary hypertension; SCAEs, serious cardiovascular adverse events; SCAEs, serious cardiovascular adverse events.

Approximately half of the pregnant women with PH experienced SCAEs (Fig. 1B, Supplementary Table 3, Supplemental Digital Content 1, http://links.lww.com/JS9/B824), primarily characterized by heart function deterioration, which was the leading cause of hospitalization. Among these patients, six individuals had serious cardiac arrhythmias, such as atrial tachycardia, atrial fibrillation, frequent premature ventricular contractions, and QTc -interval prolongation, while four patients suffered cardiac arrest. Multivariate logistic regression analysis revealed that SpO_2_ greater than 90% [odds ratio (OR) 0.05, 95% CI 0.00–0.90] and albumin level (OR 0.78, 95% CI 0.65–0.93) were protective factors, whereas estimated PAP was an independent risk factor (50–69 mmHg: OR 12.54, 95% CI 2.17–72.49; ≥70 mmHg: OR 21.03, 95% CI 2.60–170.11; Table 1).

**Table 1:** Multivariate logistic regression model showing variables independently associated with serious cardiovascular adverse events of pregnant women with PH.

Variables	OR (95% CI)	*P*
Age, year	0.98 (0.88–1.10)	0.765
BMI, kg/m^2^	1.27 (0.99–1.62)	0.056
Education level		
Never or primary education	1 (ref.)	
Middle or high school education	2.88 (0.34–24.60)	0.334
≥University education	0.37 (0.04–3.88)	0.408
Ethnicity		
Han nationality	1 (ref.)	
Ethnic minorities	0.39 (0.04–4.37)	0.448
Oxygen saturation		
SpO_2_ ≤90%	1 (ref.)	
SpO_2_ >90%	0.05 (0.00–0.90)	0.042
Albumin, g/l	0.78 (0.65–0.93)	0.006
Haemoglobin, g/l	0.99 (0.95–1.02)	0.452
PH before pregnancy		
No	1 (ref.)	
Yes	0.65 (0.17–2.48)	0.525
Ejection fraction, %	0.94 (0.87–1.03)	0.175
Eisenmenger syndrome		
No	1 (ref.)	
Yes	0.16 (0.01–2.28)	0.176
Pericardial effusion		
No	1 (ref.)	
Yes	4.50 (0.73–27.55)	0.104
Right cardiac enlargement		
No	1 (ref.)	
Yes	0.30 (0.07–1.40)	0.127
Moderate-to-severe mitral regurgitation		
No	1 (ref.)	
Yes	2.22 (0.01–567.12)	0.779
Moderate-to-severe tricuspid regurgitation		
No	1 (ref.)	
Yes	0.51 (0.10–2.49)	0.403
Estimated PAP		
36–49 mmHg	1 (ref.)	
50–69 mmHg	12.54 (2.17–72.49)	0.005
≥70 mmHg	21.03 (2.60–170.11)	0.004
Gestational weeks at delivery	0.10 (0.89–1.11)	0.928
Mode of termination of pregnancy		
Caesarean section	1 (ref.)	
Induced vaginal delivery or abortion	6.21 (0.38–101.59)	0.200
Anaesthesia method		
General anaesthesia	1 (ref.)	
Epidural anaesthesia	3.03 (0.42–21.60)	0.270
Spinal-epidural	2.41 (0.39–14.88)	0.342

OR, odds ratio; PAP, pulmonary artery pressure; PH, pulmonary hypertension; SpO2, pulse oxygen saturation.

It is crucial to establish long-term monitoring systems for ongoing follow-up and to offer comprehensive health education to pregnant women with PH, particularly those with lower levels of education and a strong desire to become pregnant. This is important because lower educational attainment may be linked to restricted access to healthcare, lower socioeconomic status, and unhealthy lifestyle habits. Screening for PH before pregnancy is advantageous for women of childbearing age with heart disease, as it allows for the early implementation of multi-stage measures to reduce PAP. Prior to delivery, accurate prediction and assessment of risks are essential to optimize management during pregnancy and postpartum. Special attention should be given to patients with moderate-to-severe PAP. Pregnant women with PH should also take steps to prevent and address hypoalbuminemia and hypoxaemia. In summary, once the decision to proceed with the pregnancy has been made, it is necessary to provide pre-pregnancy counselling, early detection, prompt referral, and comprehensive care within a specialized multidisciplinary team to prevent SCAEs and improve both maternal and foetal outcomes.

## Ethical approval

The study was ethically approved by the Ethics Committee of West China Second Hospital (No. 2023/214).

## Sources of funding

None.

## Author contribution

Y.K.: conceptualization, creation of table and figure, writing. X.J.: data acquisition, supervision.

## Conflicts of interest disclosure

The authors declare no competing interests.

## Research registration unique identifying number (UIN)

ChiCTR2300076879.

## Guarantor

All authors.

## Data statement

Data are available from the corresponding author if justification for the requirement is Justified.

## Supplementary Material

SUPPLEMENTARY MATERIAL
