# Supplementary material for: Pregnancy outcomes and risk factors of serious cardiovascular adverse events in pregnant women with pulmonary hypertension
Source: Int J Surg. 2024 Feb 8;110(3):1850–2. doi: 10.1097/JS9.0000000000001016 (PMC10942162; doi:10.1097/JS9.0000000000001016)
Supplement: SUPPLEMENTARY MATERIAL [file js9-110-1850-s001.docx]

| **Characteristics** |  |
| --- | --- |
| Age, yr, Mean (SD) | 28.5 (5.6) |
| BMI, kg/m^2^, Mean (SD) | 23.9 (3.5) |
| Ethnicity |  |
| Han nationality, n (%) | 83 (76.1) |
| Ethnic minorities, n (%) | 26 (23.9) |
| Educational level ≤ 12years, n (%) | 82 (75.2) |
| Non-compliance with visits, n (%) | 50 (45.9) |
| Multipara, n (%) | 55 (50.5) |
| Oxygen desaturation (SpO_2_ ≤90%), (n, %) | 17(15.6) |
| Albumin, g/L, Mean (SD) | 34.3 (5.7) |
| Hemoglobin, g/L, Mean (SD) | 110.9 (24.0) |
| Cardiac diagnosis, n (%) |  |
| CHD | 66 (60.6) |
| Valvular heart disease | 17 (15.6) |
| Cardiomyopathy, | 3 (2.8) |
| Idiopathic PAH or other * | 23 (21.1) |
| Cardiac diagnosis before pregnancy, n (%) | 51 (46.8) |
| PH diagnosis before pregnancy, n (%) | 44 (40.4) |

**Supplementary Table 1.** Characteristics of 109 pregnant women with PH before delivery.

* PH with unclear or multifactorial mechanisms.

Abbreviations: BMI, body mass index; CHD, congenital heart disease; PAH, pulmonary arterial hypertension; PH, pulmonary hypertension; SpO_2_, pulse oxygen saturation; SD, standard deviation;

**Supplementary Table 2.** The maternal-infant outcomes of 109 pregnant women with PH

| **Maternal outcomes** | |
| --- | --- |
| The incidence of SCAEs, n (%) | 55 (50.5) |
| ICU admission, n (%) | 91 (83.5) |
| ICU stays, days, Median (Q1, Q3) | 3.0 (2.0, 5.0) |
| LOS, days, Median (Q1, Q3) | 8.0 (6.0, 9.0) |
| **Neonatal outcomes** | |
| Fetal death >20 weeks, n (%) | 11 (10.1) |
| Neonatal death <28 days, n (%) | 1 (0.9) |
| Preterm delivery <37 weeks, n (%) | 67 (61.5) |
| Low birth weight <2500 g, n (%) | 43 (39.4) |
| NICU admission, n (%) | 44 (40.4) |

Abbreviations: ICU, intensive care unit; LOS, the length of hospital stays; NICU, neonatal intensive care unit; PH, pulmonary hypertension; SCAEs, serious cardiovascular adverse events.

**Supplementary Table 3** The comparisons of group SCAEs and No SCAEs about baseline characteristics, echocardiographic parameters and delivery management.

|  | | | SCAEs | No SCAEs | P value |
| --- | --- | --- | --- | --- | --- |
|  | | | (n = 55) | (n = 54) |  |
|  | **Baseline characteristics** |  |  |  |  |
| Age, yr, Mean (SD) | | | 28.0 (5.2) | 28.9 (5.9) | 0.426 |
| BMI, kg/m^2^, Mean (SD) | | | 24.4 (3.4) | 23.4 (3.5) | 0.125 |
| Ethnicity | | |  |  |  |
| Han nationality, n (%) | | | 38 (69.1) | 45 (83.3) | 0.081 |
| Ethnic minorities, n (%) | | | 17 (30.9) | 9 (16.7) |  |
| Educational level, n (%) | | |  |  |  |
| Never or primary education | | | 16 (29.1) | 11 (20.4) | 0.008 |
| Middle or high school education | | | 32 (58.2) | 22 (40.7) |  |
| ≥University education | | | 7 (12.7) | 21 (38.9) |  |
| Non-compliance with visits, n (%) | | | 33 (60.0) | 17 (31.5) | 0.003 |
| Parturition, n (%) | | |  |  |  |
| 0 | | | 24 (43.6) | 30 (55.6) | 0.454 |
| 1 | | | 23 (41.8) | 20 (37.0) |  |
| 2 | | | 4 (7.3) | 1 (1.9) |  |
| ≥3 | | | 4 (7.3) | 3 (5.6) |  |
| Oxygen desaturation (SpO_2_ ≤90%), n (%) | | | 15(27.3) | 2(3.7) | 0.001 |
| Albumin, g/L, Mean (SD) | | | 31.9 (5.1) | 36.7 (5.4) | <0.001 |
| Hemoglobin, g/L, Mean (SD) | | | 109.7 (26.6) | 112.1 (21.2) | 0.609 |
| Prior or gestational hypertension, n (%) | | | 7 (12.7) | 8 (14.8) | 0.752 |
| Endocrine disorders, n (%) | | | 3 (5.5) | 8 (14.8) | 0.105 |
| Autoimmune disease, n (%) | | | 2 (3.6) | 2 (3.7) | 1.000 |
| Liver disorders, n (%) | | | 4 (8.3) | 0 (0.0) | 0.052 |
| Respiratory disorders, n (%) | | | 11 (20.0) | 3 (5.6) | 0.042 |
| Kidney disorders, n (%) | | | 3 (5.5) | 1 (1.9) | 0.618 |
|  | **Echocardiographic parameters** |  |  |  |  |
| PH diagnosis before pregnancy, n (%) | | | 24 (43.6) | 20 (37.0) | 0.483 |
| Ejection fraction, %, Median (Q1, Q3) | | | 64.0 (59.0, 69.0) | 65.0 (61.0, 68.0) | 0.431 |
| Eisenmenger syndrome, n (%) | | | 11 (20.0) | 4 (7.4) | 0.056 |
| Right cardiac enlargement, n (%) | | | 32 (58.2) | 32 (59.3) | 0.909 |
| Moderate-to-severe mitral regurgitation, n (%) | | | 5 (9.1) | 1 (1.9) | 0.206 |
| Moderate-to-severe tricuspid regurgitation, n (%) | | | 13 (23.6) | 12 (22.2) | 0.861 |
| Pericardial effusion, n (%) | | | 21 (38.2) | 4 (7.4) | 0.000 |
| Estimated systolic PAP, n (%) | | |  |  |  |
| 36–49 mmHg | | | 8 (14.5) | 22 (40.7) | 0.004 |
| 50–69 mmHg | | | 19 (34.5) | 18 (33.3) |  |
| ≥70mmHg | | | 28 (50.9) | 14 (25.9) |  |
|  | **Delivery management** |  |  |  |  |
| Gestational weeks at delivery Median (Q1, Q3) | | | 34.0 (30.9, 36.0) | 35.0 (21.9, 36.9) | 0.687 |
| Mode of termination of pregnancy, n (%) | | |  |  |  |
| Caesarean section | | | 50 (90.9) | 43 (79.6) | 0.087 |
| Vaginal delivery or induced labor | | | 4 (7.3) | 7 (13.0) |  |
| Therapeutic abortion | | | 0 (0.0) | 4 (7.4) |  |
| miss data* | | | 1 (1.8) | 0 (0.0) |  |
| Mode of anesthesia, n (%) | | |  |  |  |
| General anesthesia | | | 14 (25.5) | 9 (16.7) | 0.079 |
| Epidural anesthesia | | | 20 (36.4) | 12 (22.2) |  |
| Spinal-epidural | | | 19 (34.5) | 29 (53.7) |  |

Abbreviations: BMI, body mass index; PAP, pulmonary artery pressure; SCAEs, serious cardiovascular adverse events; SpO_2_, pulse oxygen saturation; * One pregnant woman died before termination of pregnancy and was discharged at the request of her family.


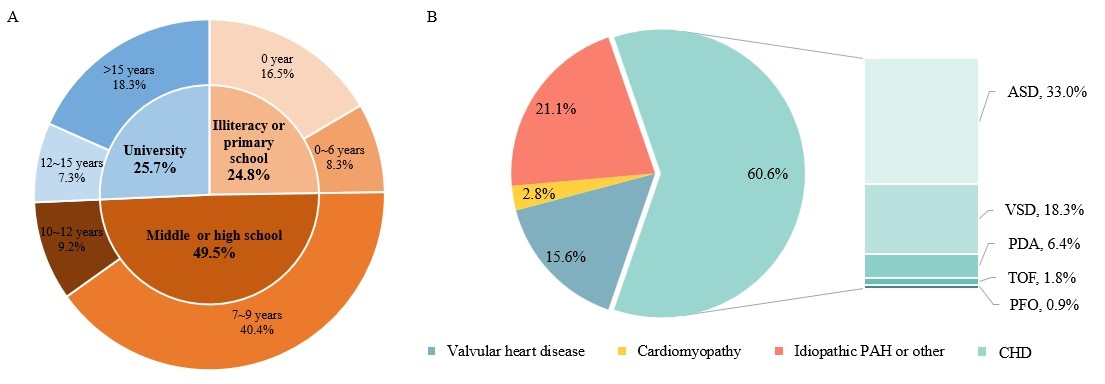
**Supplementary Figure 1.** (A) Classification of education level in pregnant women with PH. (B) Classification of heart disease in pregnant women with PH.

Abbreviations: ASD, atrial septal defect; CHD, congenital heart disease; PAH, pulmonary arterial hypertension; PDA, patent ductus arteriosus; PFO, patent foramen ovale; TOF, tetralogy of Fallot; VSD, ventricular septal defect.
